# Twelve‐Month Outcome of Nasolabial Fold Correction by a Novel Non‐1,4‐Butanediol Diglycidyl Ether, Click‐Crosslinked, Long‐Chain Hyaluronic Acid Product

**DOI:** 10.1111/jocd.70840

**Published:** 2026-04-24

**Authors:** Ari Safir, Sonja Sattler, Rodrigo Da Mota, Elzbieta Kowalska‐Olędzka, Daniel J. Kedar, Yoav Gronovich, Adi Maisel Lotan, Svetlana Shneider, Stephane Meunier, Ofir Artzi

**Affiliations:** ^1^ Division of Dermatology Tel Aviv Sourasky Medical Center Tel Aviv Israel; ^2^ Faculty of Medical & Health Sciences Tel Aviv University Tel Aviv Israel; ^3^ Rosenpark Klinik Darmstadt Germany; ^4^ Private Practice Dr. Hilton & Partner for Dermatology Düsseldorf Germany; ^5^ Maria Skłodowska‐Curie Medical Academy in Warsaw Warsaw Poland; ^6^ Evimed Warsaw Poland; ^7^ Department of Plastic and Reconstructive Surgery Sourasky Medical Center Tel Aviv Israel; ^8^ Department of Plastic and Reconstructive Surgery Shaare Zedek Medical Center Jerusalem Israel; ^9^ Faculty of Medicine Hebrew University of Jerusalem Jerusalem Israel; ^10^ Hallura R&D Yokneam Israel

**Keywords:** aesthetic medicine, click‐chemistry, hyaluronic acid, nasolabial folds, non‐BDDE crosslinking, wrinkle correction

## Abstract

**Background:**

Hyaluronic acid (HA) products used for nasolabial fold (NLF) correction are typically crosslinked with 1,4‐Butanediol diglycidyl ether (BDDE) and contain high HA concentrations and short HA chains. These characteristics may contribute to increased stiffness, enhanced immune reactivity, and less natural aesthetic outcomes. A novel HA material utilizing click‐chemistry for crosslinking to preserve long‐chain structure and afford low HA concentration and minimal modification was developed as a potential alternative.

**Aim:**

The aim of this study was to evaluate the 12‐month safety and clinical performance of a non‐BDDE, click‐chemistry crosslinked, long‐chain HA compound (HLR‐2, Hallura) for NLF correction.

**Methods:**

Two prospective, open‐label studies were conducted, one in Europe and the other in Israel. All subjects received HLR‐2 injections and were followed for 12 months. Efficacy was assessed by the Wrinkle Severity Rating Scale, Global Aesthetic Improvement Scale, and patient satisfaction measures. Safety was monitored through reporting of adverse events (AE) and documentation of injection site reactions (ISR).

**Results:**

Sixty‐two subjects (29 Israeli and 33 European) were included. Responder rates at 6 months were 90.7% and 86.4%, respectively, with improvements sustained throughout 12 months. Satisfaction levels remained high (≥ 94% at all time points). ISRs were mild and transient, with no serious or delayed‐onset AEs, and favorable induction of collagen and elastin and enhanced tissue integration were documented.

**Conclusion:**

HLR‐2 demonstrated high efficacy, high patient satisfaction, and a favorable safety profile over 12 months. The product's distinctive composition and clinical performance support its use for aesthetic indications, with potential for broader therapeutic applications.

## 1 Introduction

Hyaluronic acid (HA)‐based injectable materials are widely used in aesthetic medicine for soft tissue augmentation. Most of the commercially available products are crosslinked with 1,4‐Butanediol diglycidyl ether (BDDE) and are characterized by high HA concentrations (typically 15–26 mg/mL), short or fragmented HA chains, and significant levels of chemical modification [1, 2, 3]. These features have been associated with a higher incidence of swelling, nodule formation, delayed immune‐mediated reactions, and a less natural final aesthetic outcome [3, 4].

The 2022 Nobel Prize in Chemistry recognized the development of click‐chemistry, a biocompatible method for linking molecules under physiological conditions [5, 6]. The BiOLinkMatrix platform of Hallura applies this technology to crosslink HA without BDDE, thereby enabling minimal chemical modification (~0.9%), preservation of long‐chain HA structure, and use of lower HA concentrations (4–8 mg/mL). These features were reported to potentially result in better tissue integration, reduced inflammatory response, and a more natural aesthetic result [7, 8, 9]. This study is the first report of a 12‐month multicenter clinical evaluation of the safety and efficacy of this novel HA‐based product for nasolabial fold (NLF) correction in two cohorts.

## 2 Materials and Methods

### 2.1 Study Design

This analysis includes prospectively acquired data from two open‐label clinical studies designed to evaluate the safety and clinical performance of a non‐BDDE, click‐crosslinked HA compound characterized by a long‐chain structure and low HA concentration (HLR‐2, 6 mg/mL) for the correction of moderate‐to‐severe NLFs. One study was conducted at two sites in Germany and one in Poland (Study 19E2031; referred to as the GP cohort), and the other at three sites in Israel (Study HL01IL2022; referred to as the IL cohort). The subjects in both studies underwent treatment solely with HLR‐2. Treatments were performed by multiple experienced investigators across study sites, including seven physicians in the GP study and four physicians in the IL study, each under the supervision of the site principal investigators.

Inclusion criteria were adults aged ≥ 18 years with bilateral NLFs grade 3 or 4 on the validated Wrinkle Severity Rating Scale (WSRS) [10]. Key exclusion criteria included active dermatologic disease in the treatment area, autoimmune disorders, or recent aesthetic interventions. Pre‐defined wash‐out periods were required for prior cosmetic procedures, including temporary dermal fillers within 12 months, and botulinum toxin, mesotherapy, or energy‐based and resurfacing procedures (laser, intense pulsed light, radiofrequency, chemical peels, or dermabrasion) within 6 months prior to study entry. Additional protocol‐defined wash‐out periods for pharmacologic and dermatologic treatments were applied and are detailed in the Table S1.

### 2.2 Treatment and Follow‐Up

Injection techniques, volumes, and devices (needle or cannula) were selected at the discretion of the treating physician. All subjects were followed for at least 12 months after the last treatment.

### 2.3 Outcome Measures

Efficacy was assessed by the WSRS, with a “responder” defined as the achievement of at least a 1‐point improvement from baseline. Efficacy assessments in the IL cohort were performed by both an injector live evaluator (ILE) and a blinded live evaluator (BLE), while only the ILE was used in the GP cohort. Satisfaction and outcomes were measured by means of the Global Aesthetic Improvement Scale (GAIS) in both cohorts (a balanced 5‐point scale ranging from “much improved” to “much worse”), as rated by patients and ILE. The GP study included the FACE‐Q Appraisal of Lines module [11, 12], while the IL study included a customized Naturalness Questionnaire developed by Hallura to further assess participants' perception of aesthetic outcomes. This non‐validated tool comprised 14 statements addressing various aspects of perceived naturalness and satisfaction with treatment results. Each statement was rated on a 10‐point Likert scale ranging from 0 (“not at all”/“strongly disagree”) to 10 (“very satisfied”/“strongly agree”) (Table S2).

Safety assessments in both studies included adverse event (AE) monitoring and patient‐reported injection site reactions (ISRs) at Days 3 and 14 post‐treatment. The Israeli study incorporated additional safety measures into the protocol, including pre‐ and post‐treatment ophthalmologic and neurologic assessments to screen for rare complications, such as vascular occlusion or nerve injury.

### 2.4 Statistical Analysis

All statistical analyses were conducted by an independent statistician using SAS version 9.4 (SAS Institute, Cary, NC, USA). Unless otherwise specified, all tests were two‐tailed, and a *p*‐value ≤ 0.05 was considered statistically significant. The WSRS was used as the primary outcome measure that was assessed live by trained evaluators. NLFs were scored individually at each visit, with a “responder” defined as having achieved a ≥ 1‐grade improvement from baseline at month 6. Assuming a responder rate of 80%, a sample size of 23 subjects was estimated to yield a 95% confidence interval with a lower boundary of at least 60%. Global aesthetic improvement was evaluated by the GAIS scale, rated independently by subjects and investigators. Categorical responses were summarized as frequencies and percentages at each evaluation timepoint, and the proportion of participants reporting improvement (very much improved, much improved, or improved) was summarized accordingly.

The analysis was conducted by using three predefined populations: [1] the safety population, that included all treated subjects; [2] the full analysis set (FAS), that included all enrolled subjects with at least one post‐baseline evaluation; and [3] the per protocol (PP) population, which included all subjects with at least one post‐baseline evaluation and without any major deviation from the clinical investigation plan. Safety parameters were analyzed in the safety population, while efficacy analyses for the primary endpoint were performed for the PP population.

### 2.5 Ethical Approval

The authors confirm that the ethical policies of the journal, as noted on the journal's author guidelines page, have been adhered to and the appropriate ethical review committee approval has been received.

Both studies were approved by the relevant Institutional Review Boards (IRBs) and conducted in accordance with the Declaration of Helsinki, International Council for Harmonization (ICH) Good Clinical Practice (GCP) guidelines, ISO 14155:2020, and local regulatory requirements.

The GP study (Protocol 19E2031) received ethical approval from appropriate local ethics committees in accordance with EU MDR (Regulation (EU) 2017/745). The study was initiated on October 26, 2020, and completed on September 6, 2022. It was prospectively registered at ClinicalTrials.gov (Identifier: NCT04971876). All participants gave written informed consent before undergoing any study‐related procedures.

The IL study (Protocol HL01IL202) received IRB approval from participating institutions including Shaare Zedek Medical Center and Ichilov Sourasky Medical Center. The study was initiated on January 9, 2023, and continued through February 2024, with ethical approval granted prior to the enrollment of the first participant. Ethics oversight was provided at each of the six participating clinical sites. All subjects provided written informed consent prior to any study‐related procedures.

## 3 Results

### 3.1 Demographic Characteristics

A total of 62 subjects were enrolled—33 in GP and 29 in IL—to evaluate the performance and safety of HLR‐2 for nasolabial fold correction. In the IL cohort, 29 subjects were enrolled, of whom 27 reached the primary endpoint at 6 months and were therefore included in the performance analysis. As nasolabial folds were evaluated bilaterally, these 27 subjects corresponded to 54 treated hemifaces, which formed the basis for the performance and responder rate analyses (Sections 3.2 and 3.3). The demographic characteristics of both cohorts were similar (Table 1). Most of the participants were females who had Fitzpatrick skin types I–III, with darker skin types (IV–V) being infrequent.

**TABLE 1:** Baseline demographic and clinical characteristics of enrolled subjects by study site.

Characteristic	IL study (*n* = 29)	GP study (*n* = 33)
Mean age (years)	47.7 ± 8.9	50.7 ± 6.9
Female	28 (96.6%)	30 (90.9%)
Fitzpatrick I–III	28 (97%)	30 (91%)
Fitzpatrick IV–V	1 (3%)	3 (9%)
WSRS scores at enrolment (% of PP population)	3 (75.9%)	3 (68.8%)
	4 (24.1%)	4 (31.2%)

*Note:* WRSR evaluation by Evaluator (BLE) in the Israeli study (HL01IL2022), and by ILE in the European study (19E2031).

Abbreviations: GP, Germany and Poland site study; IL, Israel site study; PP, per protocol population; WSRS, wrinkle severity rating scale.

### 3.2 Baseline Severity and Treatment Characteristics

All of the subjects in both cohorts presented with moderate‐to‐severe NLFs, as defined by a WSRS score of 3 or 4 at baseline (Table 1). Most of the NLFs (75.9%) in the IL cohort were graded WSRS 3 while 24.1% were graded WSRS 4. The respective distribution in the GP cohort was 68.8% and 31.2%. The difference in baseline WSRS scores between the two cohorts was not significant (*p* = 0.485), indicating comparable pre‐treatment severity.

There were notable differences in injection regimens between the two study cohorts. Initial treatment volumes per nasolabial fold were comparable, with a mean of 1.66 ± 0.4 mL in the IL cohort and 1.40 ± 0.7 mL in the GP cohort. Optional touch‐up treatments were administered in 31.5% of NLFs in the IL cohort (17/54 hemifaces), with a mean touch‐up volume of 0.8 mL per hemiface (range 0.4–1.5 mL). In the GP cohort, touch‐up treatments were administered in a higher proportion of NLFs (97.0%, 64/66 hemifaces), with a mean touch‐up injection volume of 1.5 mL per hemiface (range 0.35–3.0 mL).

Injection techniques were selected at the discretion of the treating physician and varied notably between cohorts. In the IL study, nearly all of the injections were performed with needles (93% in initial injection and 100% in touch‐up), with “linear threading retrograde” and “bolus” being the most commonly used techniques. The GP study had greater technique diversity, with frequent use of both needles and cannulas, as well as a higher prevalence of combination methods, such as layering, fanning, and depot injections. No injection device malfunctions were reported in either study.

### 3.3 Performance Outcomes—Responder Rates

The primary endpoint was defined as the proportion of nasolabial folds (NLFs) achieving a ≥ 1‐grade improvement on the WSRS scale at 6 months post‐treatment.

In the IL cohort, 27 subjects (54 NLFs) reached the primary endpoint visit and were included in the performance analysis. Of these, 24 subjects were responders on both NLFs, one subject was a responder on one NLF only, resulting in 49/54 NLFs (90.7%) meeting the primary endpoint when assessed by the investigator live evaluator (ILE). Assessment by the blinded live evaluator (BLE) yielded a comparable responder rate of 50/54 NLFs (92.6%). Mean WSRS improvements were 1.65 ± 0.76 and 1.43 ± 0.74, respectively (both *p* < 0.001).

In the GP cohort, all 33 subjects attended the primary endpoint visit at 6 months. Twenty‐eight subjects were responders on both NLFs, and one subject was a responder on one NLF only, resulting in 57/66 treated NLFs (86.4%) achieving the primary endpoint, with a mean WSRS improvement of 1.42 ± 0.59 (*p* < 0.001).

**FIGURE 1:**
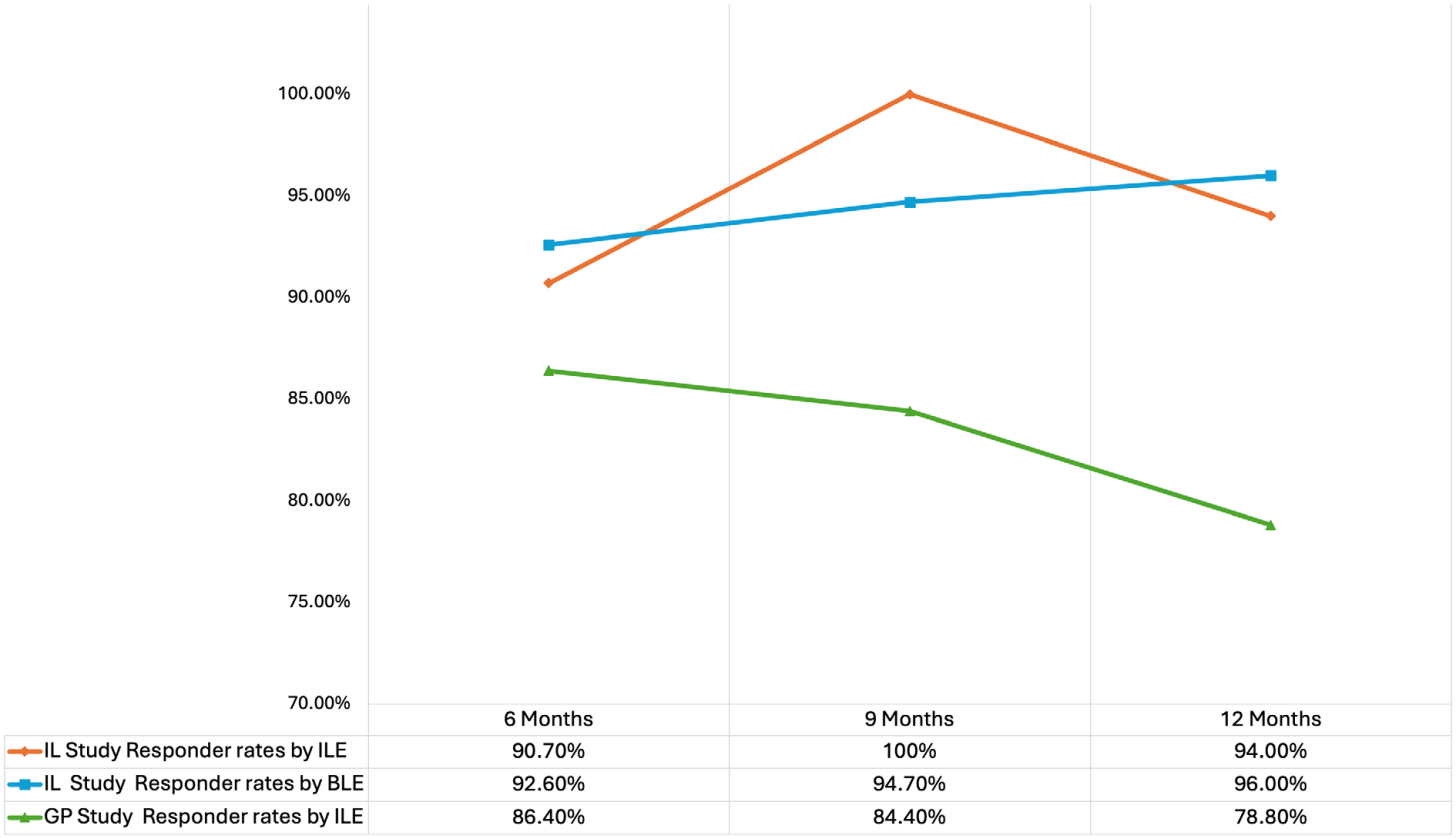
HLR‐2 responder rates across cohorts and follow‐up periods. The graph illustrates the percentage of subjects achieving a ≥ 1 grade improvement on the WSRS scale at 6, 9, and 12 months post‐treatment in each of two clinical cohorts. The lines represent responder rates as assessed by Investigator Live Evaluator (ILE) and Blinded Live Evaluator (BLE) in the Israeli (IL) study, and by ILE in the German‐Poland (GP) study.

The responder rates remained high throughout 12 months in both studies, with the IL cohort maintaining a rate ≥ 94.0% and the GP cohort a rate of ≥ 78.8%. For contextual reference, objective 12‐month responder rates reported for established BDDE‐crosslinked hyaluronic acid fillers approved for nasolabial fold correction generally range from approximately 50%–58%, based on investigator‐ or blinded‐evaluator–assessed WSRS/NLF severity scale outcomes [13, 14]. These results reflect durable clinical performance across evaluators and study populations (Figure 1).

### 3.4 Subject‐Reported Outcomes

Aesthetic satisfaction was consistently high for both patient populations. The vast majority of subjects and physicians rated outcomes on the GAIS as “improved” or “much improved” at all time points. Almost all (95%–100%) of the IL subjects and physicians (ILE) reported improvement with full agreement at 12 months. Likewise, the subject‐rated improvement remained at 100% throughout 9 months and 97% at 12 months, with similarly high ratings (97% at 6 months and 94% at 12 months) among the GP subjects and physicians (Table S3).

In addition, the FACE‐Q Appraisal of Lines module that was used in the GP study demonstrated significant improvement in satisfaction from baseline as early as two weeks post‐treatment (*p* < 0.001), with benefits maintained throughout the ensuing 12 months.

The IL participants were administered a custom‐designed satisfaction and naturalness questionnaire at the 12‐month follow‐up. A total of 25 subjects completed the questionnaire. Notably, 100% of subjects (*N* = 25) reported that the aesthetic result appeared natural, and 100% of respondents stated they would undergo the treatment again and recommend it to others (Table S2).

### 3.5 Safety Outcomes

Both the IL and GP studies demonstrated a favorable safety profile for HLR‐2, with no serious adverse events (SAEs) or unanticipated negative device‐related events reported throughout the study period. Two mild AEs (dizziness in one Israeli patient and headache in another Israeli patient) were considered possibly related to treatment, and both resolved spontaneously without medical intervention. The Israeli study also included enhanced safety monitoring protocols, such as pre‐treatment ophthalmologic examinations and neurological assessments, and no ocular or neurologic adverse effects were observed at any time during the 12‐month follow‐up.

**FIGURE 2:**
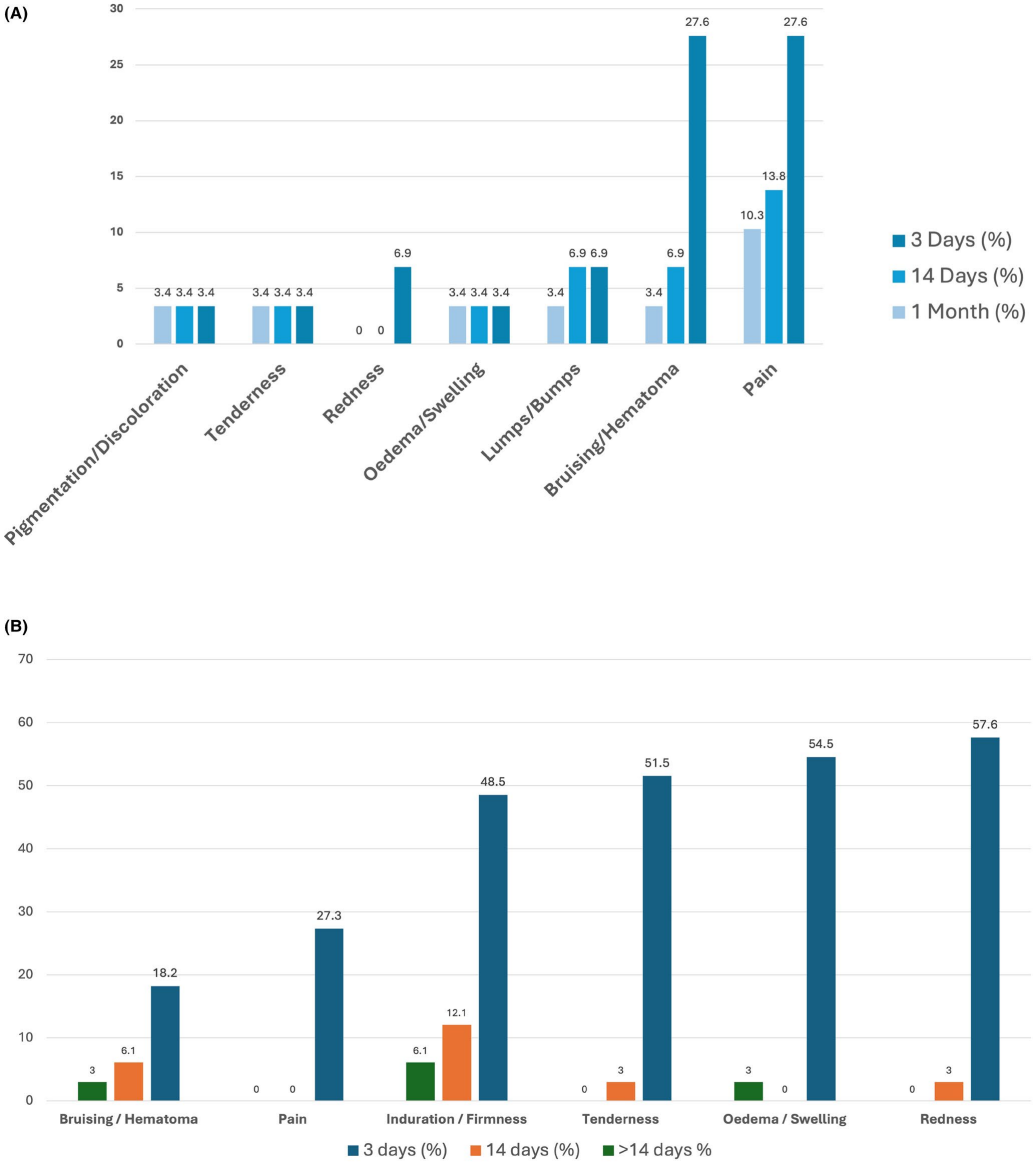
Incidence of injection site reactions over time. (A) Israeli cohort (IL study, *N* = 29 subjects): Data represent the percentage of subjects reporting each ISR at each timepoint. (B) German–Polish cohort (GP study, *N* = 33 subjects): Data represent the percentage of subjects reporting each injection‐site reaction (ISR) at each timepoint.

The most common ISRs reported by Israeli subjects 3 days post‐injection were bruising/hematoma and pain, each observed in 8 out of 29 subjects (27.6%), and mostly classified as mild (75% and 62.5%, respectively). Additional ISRs included lumps/bumps (6.9%, 2/29), oedema/swelling (3.4%, 1/29), redness (6.9%, 2/29), tenderness (3.4%, 1/29), and pigmentation/discoloration (3.4%, 1/29), all of which were mild or moderate. The total number of ISRs decreased from 22 events at 3 days to 13 at 14 days and 8 at 1 month (Figure 2A). No severe ISRs were reported at any time point.

In the GP cohort, the most common ISRs reported within the first 3 days post‐injection were redness (19/33 subjects; 57.6%), oedema/swelling (18/33; 54.5%), induration/firmness (16/33; 48.5%), and tenderness 17/33; 51.5. Most of these reactions were classified as mild, with moderate events occurring less frequently, ranging from 4.6% to 22.7%, and severe reactions rarely reported, not exceeding 7.6% for any reaction. Most ISRs occurred within the first 3 days post‐injection and showed improvement during the first 14 days in most cases (Figure 2B). No ISR persisted beyond 30 days, and all resolved spontaneously without sequelae or the need for treatment. No delayed‐onset inflammatory nodules, vascular events, or hypersensitivity reactions were reported over the 12‐month observation period.

## 4 Discussion

The global use of HA‐based injections has increased substantially over the past two decades, extending beyond aesthetic wrinkle correction to include therapeutic and dermatologic indications [15, 16]. This clinical and mainly aesthetic expansion has coincided with significant market growth, with the global HA market projected to reach around USD 16.8 billion by 2030 [17]. This growth has been accompanied both by a shift in aesthetic preferences toward a more natural outcome in facial structure and expression, with patient and physician expectations increasingly favoring treatments that offer subtle natural enhancement [18, 19]; and by an increased incidence of complications, including swelling, nodules, delayed immune responses, or “overfilled” or unnatural appearance [18, 20, 21].

Contributing factors to these less desirable outcomes include injection technique, patient‐specific characteristics (e.g., age, skin quality, anatomical variation, or immune reactivity) and the characteristics of the HA product used [22]. Currently available HA products are predominantly crosslinked with BDDE, a chemical agent that enables extended persistence in tissue. These formulations typically feature high HA concentrations (15–26 mg/mL), short or fragmented HA chains, and a high degree of crosslinking [1, 4, 9]. Such properties can result in increased stiffness, a tendency toward excessive water absorption (leading to swelling), and a higher risk of immune‐mediated reactions. Additionally, BDDE‐crosslinked products raise concerns of potential residual toxicity, carcinogenicity, and the formation of uncharacterized by‐products during manufacturing [3, 23]. Furthermore, BDDE degradation can produce pro‐inflammatory low molecular weight HA fragments that may interact with toll‐like receptors and contribute to adverse immune responses [4, 24, 25]. Taken together, these considerations have led to an increasing interest in developing new HA materials with improved biocompatibility, different rheology, and reduced risk of AEs.

Click‐chemistry is a bio‐orthogonal approach that enables the formation of stable covalent bonds under physiological conditions without generating reactive byproducts. Originally developed for chemical biology, this method has since been applied in various biomedical domains, including controlled drug delivery systems, implantable hydrogels, antibody‐drug conjugates, and in vivo imaging tools [24, 26, 27, 28]. These applications support its clinical relevance due to their high specificity, low toxicity, and compatibility with living systems. These characteristics motivated the exploration of click‐chemistry as a novel crosslinking strategy for HA, with the goal of creating a material that maintains structural integrity while offering improved integration and tolerability in soft tissue applications. This study presents, for what we believe to be the first time, clinical data on the use of a non‐BDDE, click‐chemistry crosslinked HA compound for aesthetic applications.

The product demonstrated consistent and sustained performance for the correction of moderate‐to‐severe nasolabial folds (NLFs) over a 12‐month follow‐up period in independent analyses conducted in Israel, Germany and Poland. The results were comparable or superior to other tissue fillers: in a large meta‐analysis by Stefura et al. [7], which included 51 randomized clinical trials involving 4097 patients, a pooled WSRS reduction of approximately 1.21 points at 6 months (from 3.23 to 2.02) and 0.77 points at 12 months (from 3.23 to 2.46) was reported. In comparison, the click‐crosslinked HA product evaluated herein achieved a mean WSRS improvement of 1.65 in the Israeli cohort and 1.42 in the German cohort at 6 months.

**FIGURE 3:**
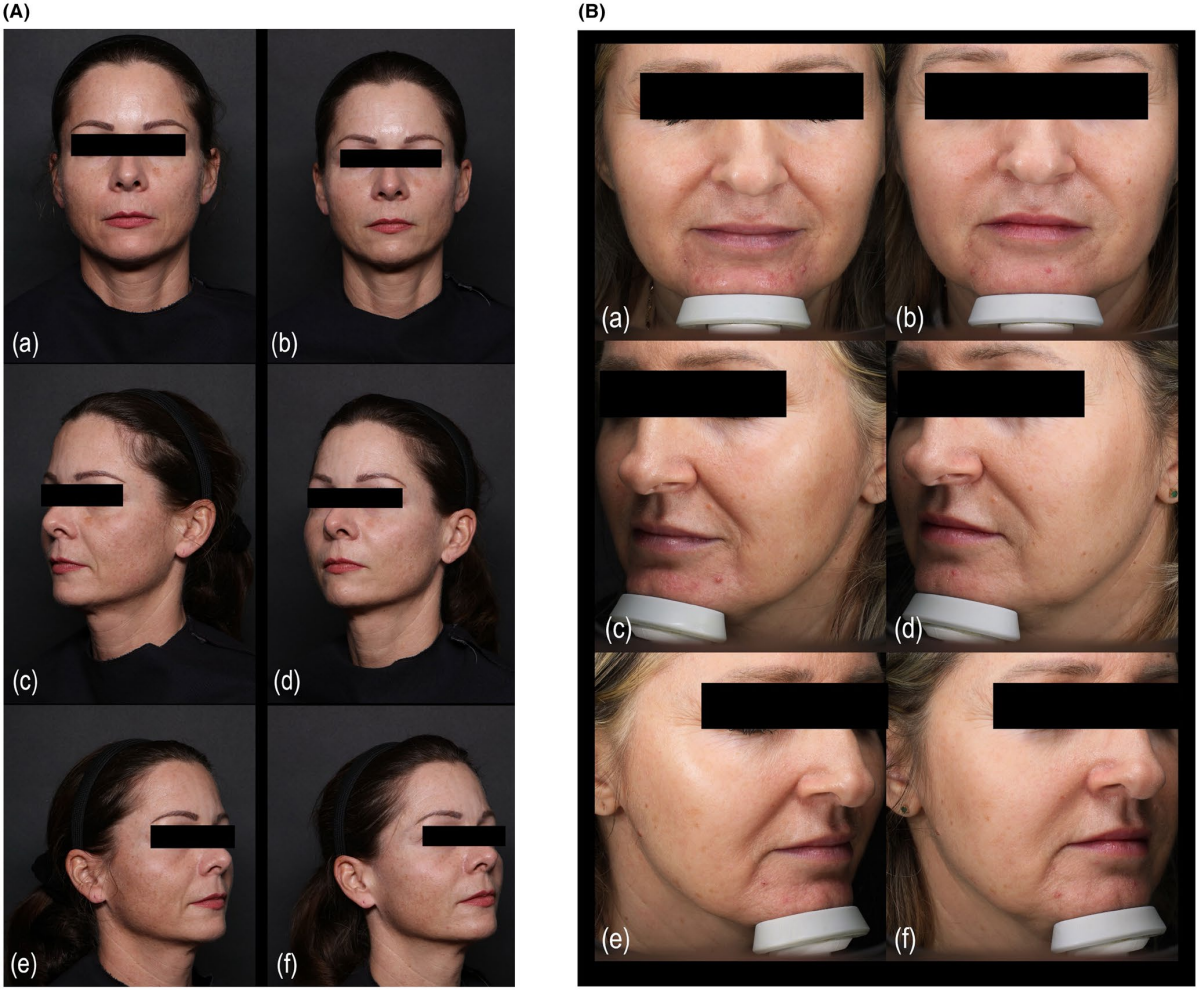
Clinical results following HLR‐2 injection for nasolabial fold correction. Representative subject from the German and Poland cohort (A) and from Israeli cohort (B) demonstrating natural aesthetic improvement 12 months after treatment with HLR‐2 for nasolabial folds. Left column (a, c, e): Baseline images (frontal, left oblique, right oblique). Right column (b, d, f): Corresponding post‐treatment views at 12 months.

Responder rates, defined as the proportion of nasolabial folds with a ≥ 1‐grade improvement on the WSRS, ranged from 86% to 90% across both cohorts and were sustained throughout 12 months since last treatment. These successful clinical outcomes were further supported by high levels of patient satisfaction and self‐reported natural aesthetic appearance, particularly in the Israeli cohort, where 100% of the participants reported the results as being natural and age appropriate (Figure 3). We consider that these descriptions reflect a growing preference for results in aesthetic medicine that are objectively and subjectively effective while being subtle and otherwise inconspicuous.

Interestingly, comparable efficacy outcomes were observed between the IL and GP cohorts despite notable differences in injection regimens, particularly in the frequency of touch‐up treatments and injected volumes. The achievement of similar clinical efficacy in the IL cohort with substantially fewer and lower‐volume touch‐up treatments suggests that the product may provide sustained clinical benefit. This divergence may, at least in part, reflect the evolutionary nature of clinical experience with this novel formulation. As a first‐in‐human study, physicians in the GP cohort more frequently employed a second injection as a conservative strategy when working with an investigational product whose in vivo behavior and durability are not yet fully characterized.

From a safety perspective, ISRs observed in both cohorts were predominantly mild and transient. While no direct safety comparison with conventional cross‐linked HA fillers was performed, published randomized and controlled studies of BDDE‐crosslinked HA fillers for nasolabial folds report early ISRs such as erythema, swelling, tenderness, lumps, or induration have been reported in approximately 30%–80% of treated subjects, typically resolving within 2–14 days. Longer term randomized trials have similarly reported self‐limiting injection‐site swelling, redness, and tenderness in approximately 10%–30% of subjects [29, 30, 31]. Late‐onset inflammatory nodules and granulomatous reactions are rare (reported in roughly 0.5%–4% with specific formulations) and typically appear several weeks to months after treatment [20, 21]; no such events were documented with HLR‐2 during the follow‐up period. Taken together, these data suggest that the safety outcomes observed with HLR‐2 fall within the expected range. Nevertheless, given the relatively limited sample size of the present study, larger and longer term investigations are warranted to more fully characterize rare or late‐onset adverse events.

The molecular weight of HA plays a critical role in determining its biological behavior. Long‐chain, high molecular weight HA, typically exceeding 1000 kDa, is primarily associated with anti‐inflammatory, anti‐proliferative, and anti‐angiogenic effects, contributing to tissue homeostasis and regenerative balance [32, 33, 34]. HA concentrations also have an important impact: lower concentrations of HA, particularly in minimally modified or non‐crosslinked forms, have been shown to effectively engage cellular receptors and promote stem cell activity without overwhelming the extracellular environment or triggering adverse responses [35]. Therefore, maintaining minimal chemical modification is essential to preserving HA's intrinsic bioactivity and its ability to engage receptors, such as CD44, as this interaction supports anti‐inflammatory effects, cellular rest states, and regenerative functions [34].

Hallura's BiOLinkMatrix platform applies this technology to crosslink HA without BDDE, thereby enabling minimal chemical modification (~0.9%), preservation of long‐chain HA structure, and use of lower HA concentrations. These physicochemical features may endow the product with positive biological benefits. Immunohistochemical studies have been conducted to evaluate the biological effects of HLR products, including HLR‐2. An ex vivo human skin explant study demonstrated stimulated growth of elastin and collagen I networks within 7 days post‐injection (Figure S1). These observations were supported by an in vitro fibroblast study comparing HLR‐2 to a leading BDDE‐based filler in which HLR‐2 demonstrated superior production of collagen I, collagen III, and elastin. Histopathological evaluations in rats demonstrated excellent tissue integration, a better physiological tissue response, and favorable natural tissue ingrowth compared to commercial products (Figure S2), with significantly lower fibrous capsule thickness (Table S4).

## 5 Conclusions

The data presented in this study support the clinical relevance of a novel, non‐BDDE, click‐chemistry crosslinked HA formulation for the correction of nasolabial folds. The product demonstrated high efficacy, sustained clinical outcomes over 12 months, and excellent patient‐reported satisfaction. In addition, its physicochemical properties, along with findings from preclinical histological and immunohistochemical studies, suggest potential bioregenerative activity. If substantiated, this could position the product within a new category of crosslinked HA materials suitable not only for conventional aesthetic indications—such as volumization, wrinkle correction, tear trough filling, and skin quality improvement—but also for emerging therapeutic applications. Further clinical investigations are warranted to elucidate the specific biomechanisms of action and to define the full scope of clinical applications. Future studies will be essential to confirm the current findings and to explore broader aesthetic and therapeutic applications.

## Author Contributions

Ari Safir – data analysis, writing original draft, review and editing. Sonja Sattler – investigation, supervision, writing – review and editing. Rodrigo Da Mota, Elzbieta Kowalska‐Olędzka, Daniel J. Kedar, and Yoav Gronovich – inestigation, supervision. Sveta Shneider and Stephane Meunier – project administration, conceptualization, and resources. Ofir Artzi – conceptualization, investigation, writing – original draft, review, and editing.

## Funding

The studies were funded by Hallura Ltd. The authors received no honorarium/fee or other forms of financial support related to the development of this article.

## Ethics Statement

The authors confirm that the ethical policies of the journal, as noted on the journal's author guidelines page, have been adhered to and the appropriate ethical review committee approval has been received. This manuscript includes data from two prospective clinical studies, both conducted in accordance with the Declaration of Helsinki, ICH Good Clinical Practice (GCP) guidelines, ISO 14155:2020, and applicable local regulations. Protocol 19E2031 received approval from local ethics committees in Europe in accordance with EU MDR (Regulation (EU) 2017/745), with study initiation on October 26, 2020. Protocol HL01IL202 was approved by institutional review boards of all participating Israeli centers, including Shaare Zedek Medical Center and Tel Aviv Sourasky Medical Center (Ichilov), with study initiation on January 9, 2023. All participants provided written informed consent prior to any study‐related procedures. Both studies were prospectively registered at ClinicalTrials.gov (Identifier: NCT04971876).

## Conflicts of Interest

S. Shneider and S. Meunier are employees of Hallura Ltd. The opinions expressed in this article are those of the authors. The authors received no honorarium/fee or other forms of financial support related to the development of this article. Dr. Sonja Sattler is a paid consultant, speaker, researcher, and global KOL for MERZ AESTHETICS, speaking and conducting clinical research for Hallura, Crown Aesthetics, and ICA Navigation System GmbH, and is conducting clinical research for Abbvie, Allergan Aesthetics, LG, CROMA, IPSEN, and Evolus.

## Supporting information


**Figure S1:** Natural tissue biostimulation by Hallura's HA. This figure demonstrates the enhancement of elastin networks by Hallura's HA product. Representative immunostaining images illustrating (a) bio‐stimulated elastin (green) in non‐treated skin and (b) 7 days post‐treatment.

**Figure S2:** Histological evaluation at 13 weeks following subdermal administration of different types of hyaluronic acids. Representative histological images from a 13‐week subdermal implantation study in Sprague–Dawley rats comparing two commercial hyaluronic acid (HA) fillers (a, b, e, f) and the investigational HLR product (c, g). Top row (a–c): Hematoxylin and eosin (H&E) staining shows tissue architecture and capsule formation around the injected materials. Bottom row (e–g): Picrosirius Red staining under polarized light highlights collagen distribution (types I and III). HLR (c, g) demonstrated superior tissue integration and dispersed collagen deposition within the gel.

**Table S1:** Detailed Exclusion criteria and wash‐out periods.
**Table S2:** Satisfaction, naturalness questionnaire.
**Table S3:** Satisfaction, GAIS (% of responders as improved or much improved).
**Table S4:** Summary of manufacturer‐provided preclinical biological and tissue‐level evaluation of Hallura products, including rheologic characteristics.

## Data Availability

The data that support the findings of this study are available from the corresponding author upon reasonable request.

## References

[1] J. Faivre , A. I. Pigweh , J. Iehl , et al., “Crosslinking Hyaluronic Acid Soft‐Tissue Materials: Current Status and Perspectives From an Industrial Point of View,” Expert Review of Medical Devices 18, no. 12 (2021): 1175–1187. 34882503 10.1080/17434440.2021.2014320

[2] S. Wang , S. Tavakoli , R. P. Parvathaneni , et al., “Dynamic Covalent Crosslinked Hyaluronic Acid Hydrogels and Nanomaterials for Biomedical Applications,” Biomaterials Science 10, no. 22 (2022): 6399–6412. 36214100 10.1039/d2bm01154a

[3] M. Wojtkiewicz , A. I. Stachura , B. Roszkowski , et al., “Are we Overlooking Harms of BDDE‐Cross‐Linked Dermal Fillers? A Scoping Review,” Aesthetic Plastic Surgery 48, no. 23 (2024): 5147–5154. 39107664 10.1007/s00266-024-04262-0 PMC11739315

[4] W. Lee , S. Shah‐Desai , N. K. Rho , and J. Cho , “Etiology of Delayed Inflammatory Reaction Induced by Hyaluronic Acid Filler,” Archives of Plastic Surgery 51, no. 1 (2024): 20–26. 38425859 10.1055/a-2184-6554 PMC10901605

[5] C. M. Madl and S. C. Heilshorn , “Bioorthogonal Strategies for Engineering Extracellular Matrices,” Advanced Functional Materials 28, no. 11 (2018): 1706046–1706067. 31558890 10.1002/adfm.201706046 PMC6761700

[6] C. R. Bertozzi , “A Decade of Bioorthogonal Chemistry,” Accounts of Chemical Research 44, no. 9 (2011): 651–653. 21928847 10.1021/ar200193f PMC4408923

[7] T. Stefura , A. Kacprzyk , J. Droś , et al., “Tissue Fillers for the Nasolabial Fold Area: A Systematic Review and Meta‐Analysis of Randomized Clinical Trials,” Aesthetic Plastic Surgery 45, no. 5 (2021): 2300–2316. 34255156 10.1007/s00266-021-02439-5 PMC8481177

[8] P. Snozzi and J. van Loghem , “Clinical Implications of Delayed‐Onset Inflammatory Reactions to Hyaluronic Acid Fillers: A Review,” Clinical, Cosmetic and Investigational Dermatology 11 (2018): 277–283.

[9] J. Kablik , G. D. Monheit , L. Yu , G. Chang , and J. Gershkovich , “Comparative Physical Properties of Hyaluronic Acid Dermal Fillers,” Dermatologic Surgery 35, no. Suppl 1 (2009): 302–312. 19207319 10.1111/j.1524-4725.2008.01046.x

[10] D. J. Day , C. M. Littler , R. W. Swift , and S. Gottlieb , “The Wrinkle Severity Rating Scale: A Validation Study,” American Journal of Clinical Dermatology 5, no. 1 (2004): 49–52. 14979743 10.2165/00128071-200405010-00007

[11] A. F. Klassen , S. J. Cano , A. Scott , L. Snell , and A. L. Pusic , “Measuring Patient‐Reported Outcomes in Facial Aesthetic Patients: Development of the FACE‐Q,” Facial Plastic Surgery 26, no. 4 (2010): 303–309. 20665408 10.1055/s-0030-1262313

[12] A. F. Klassen , S. Cano , J. Mansouri , et al., ““I Want It to Look Natural”: Development and Validation of the FACE‐Q Aesthetics Natural Module,” Aesthetic Surgery Journal 44, no. 7 (2024): 733–743. 38180487 10.1093/asj/sjad374 PMC11177552

[13] Administration USFaD , “Summary of Safety and Effectiveness Data (SSED) for Juvéderm Vollure XC,” (Center for Devices and Radiological Health (CDRH), U.S. Food and Drug Administration, 2017). Report No.: P110033/S020.

[14] Y. Xie , W. Wu , J. Xu , X. Wang , Z. Hu , and Q. Li , “A Randomized, Multicenter Study on a Flexible Hyaluronic Acid Filler in Treatment of Moderate‐To‐Severe Nasolabial Folds in a Chinese Population,” Journal of Cosmetic Dermatology 21, no. 10 (2022): 4288–4293. 35279948 10.1111/jocd.14914

[15] D. Funt and T. Pavicic , “Dermal Fillers in Aesthetics: An Overview of Adverse Events and Treatment Approaches,” Clinical, Cosmetic and Investigational Dermatology 6 (2013): 295–316. 24363560 10.2147/CCID.S50546 PMC3865975

[16] S. K. Thareja , D. Sadhwani , and F. N. Alan , “En Coup de Sabre Morphea Treated with Hyaluronic Acid Filler. Report of a Case and Review of the Literature,” International Journal of Dermatology 54, no. 7 (2015): 823–826. 24168261 10.1111/ijd.12108

[17] J. E. Ryu , J.‐M. Kim , and B.‐Y. Kim , “Hyaluronic Acid: A Powerful Biomolecule With Wide‐Ranging Applications,” International Journal of Molecular Sciences 24, no. 6 (2023): 301. 10.3390/ijms2406301. PMC10299688 37373443

[18] N. Corduff , L. Juniarti , T. S. Lim , et al., “Current Practices in Hyaluronic Acid Dermal Filler Treatment in Asia Pacific and Practical Approaches to Achieving Safe and Natural‐Looking Results,” Clinical, Cosmetic & Investigational Dermatology 15 (2022): 1213–1223. 35800454 10.2147/CCID.S363583 PMC9255720

[19] M. Barone , R. De Bernardis , and P. Persichetti , “Aesthetic Medicine Across Generations: Evolving Trends and Influences,” Aesthetic Plastic Surgery 49 (2024): 3274–3276. 39227469 10.1007/s00266-024-04353-y

[20] W. Baranska‐Rybak , J. V. Lajo‐Plaza , L. Walker , and N. Alizadeh , “Late‐Onset Reactions After Hyaluronic Acid Dermal Fillers: A Consensus Recommendation on Etiology, Prevention and Management,” Dermatology and Therapy (Heidelberg) 14, no. 7 (2024): 1767–1785. 10.1007/s13555-024-01202-3 PMC11265052 38907876

[21] O. Artzi , J. L. Cohen , J. S. Dover , et al., “Delayed Inflammatory Reactions to Hyaluronic Acid Fillers: A Literature Review and Proposed Treatment Algorithm,” Clinical, Cosmetic & Investigational Dermatology 13 (2020): 371–378. 32547150 10.2147/CCID.S247171 PMC7244356

[22] G. W. Hong , J. Wan , Y. Park , et al., “Rheological Characteristics of Hyaluronic Acid Fillers as Viscoelastic Substances,” Polymers (Basel) 16, no. 16 (2024): 2386. 39204605 10.3390/polym16162386 PMC11360814

[23] M. Litwiniuk , A. Krejner , M. S. Speyrer , A. R. Gauto , and T. Grzela , “Hyaluronic Acid in Inflammation and Tissue Regeneration,” Wounds 28, no. 3 (2016): 78–88. 26978861

[24] D. H. Kim , C. H. Jeong , J. H. Han , et al., “Comparative Toxicity Study of Hyaluronic Acid Fillers Crosslinked With 1,4‐Butanediol Diglycidyl Ether or Poly (Ethylene Glycol) Diglycidyl Ether,” International Journal of Biological Macromolecules 296 (2025): 139620. 39788250 10.1016/j.ijbiomac.2025.139620

[25] K. R. Taylor , J. M. Trowbridge , J. A. Rudisill , C. C. Termeer , J. C. Simon , and R. L. Gallo , “Hyaluronan Fragments Stimulate Endothelial Recognition of Injury Through TLR4,” Journal of Biological Chemistry 279, no. 17 (2004): 17079–17084, 10.1074/jbc.M310859200. 14764599

[26] K. Nwe and M. W. Brechbiel , “Growing Applications of “Click Chemistry” for Bioconjugation in Contemporary Biomedical Research,” Cancer Biotherapy & Radiopharmaceuticals 24, no. 3 (2009): 289–302. 19538051 10.1089/cbr.2008.0626 PMC2811415

[27] A. Battigelli , B. Almeida , and A. Shukla , “Recent Advances in Bioorthogonal Click Chemistry for Biomedical Applications,” Bioconjugate Chemistry 33, no. 2 (2022): 263–271. 35107252 10.1021/acs.bioconjchem.1c00564

[28] M. R. Arkenberg , H. D. Nguyen , and C. C. Lin , “Recent Advances in Bio‐Orthogonal and Dynamic Crosslinking of Biomimetic Hydrogels,” Journal of Materials Chemistry B 8, no. 35 (2020): 7835–7855. 32692329 10.1039/d0tb01429j PMC7574327

[29] J. Colon , S. Mirkin , P. Hardigan , M. J. Elias , and R. J. Jacobs , “Adverse Events Reported From Hyaluronic Acid Dermal Filler Injections to the Facial Region: A Systematic Review and Meta‐Analysis,” Cureus 15, no. 4 (2023): e38286. 37261136 10.7759/cureus.38286 PMC10226824

[30] L. N. Trinh and A. Gupta , “Hyaluronic Acid Fillers for Midface Augmentation: A Systematic Review,” Facial Plastic Surgery 37, no. 5 (2021): 576–584. 33634456 10.1055/s-0041-1724122

[31] Y. Hu , Z. Liu , Y. Wang , et al., “Efficacy and Safety of Two Hyaluronic Acid Fillers With Different Injection Depths for the Correction of Moderate‐To‐Severe Nasolabial Folds: A 52‐Week, Prospective, Randomized, Double‐Blinded Study in a Chinese Population,” Journal of Cosmetic Dermatology 21, no. 3 (2022): 940–948. 35020250 10.1111/jocd.14744

[32] A. D'Agostino , A. Stellavato , L. Corsuto , et al., “Is Molecular Size a Discriminating Factor in Hyaluronan Interaction With Human Cells?,” Carbohydrate Polymers 157 (2017): 21–30. 27987920 10.1016/j.carbpol.2016.07.125

[33] Y. Kawano , V. Patrulea , E. Sublet , et al., “Wound Healing Promotion by Hyaluronic Acid: Effect of Molecular Weight on Gene Expression and in Vivo Wound Closure,” Pharmaceuticals (Basel) 14, no. 4 (2021): 301. 33800588 10.3390/ph14040301 PMC8065935

[34] A. G. Tavianatou , I. Caon , M. Franchi , Z. Piperigkou , D. Galesso , and N. K. Karamanos , “Hyaluronan: Molecular Size‐Dependent Signaling and Biological Functions in Inflammation and Cancer,” FEBS Journal 286, no. 15 (2019): 2883–2908. 30724463 10.1111/febs.14777

[35] N. Alessio , A. Stellavato , T. Squillaro , et al., “Hybrid Complexes of High and Low Molecular Weight Hyaluronan Delay in Vitro Replicative Senescence of Mesenchymal Stromal Cells: A Pilot Study for Future Therapeutic Application,” Aging (Albany NY) 10, no. 7 (2018): 1575–1585. 30001217 10.18632/aging.101493 PMC6075440

