# Supplementary figures and images for: Twelve‐Month Outcome of Nasolabial Fold Correction by a Novel Non‐1,4‐Butanediol Diglycidyl Ether, Click‐Crosslinked, Long‐Chain Hyaluronic Acid Product
Source: J Cosmet Dermatol. 2026 Apr 24;25(4):e70840. doi: 10.1111/jocd.70840 (PMC13108564; doi:10.1111/jocd.70840)

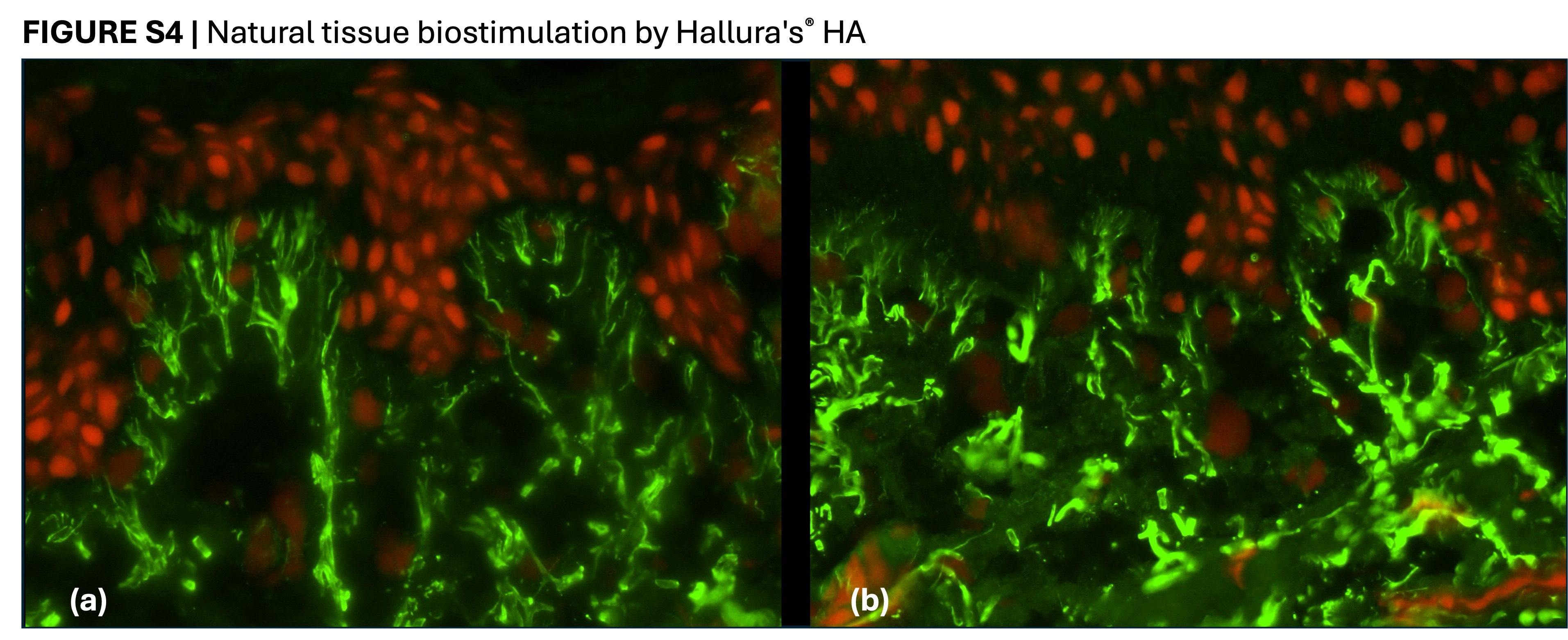

Supplement: Supplementary file 1 — Figure S1: Natural tissue biostimulation by Hallura's HA. This figure demonstrates the enhancement of elastin networks by Hallura's HA product. Representative immunostaining images illustrating (a) bio‐stimulated elastin (green) in non‐treated skin and (b) 7 days post‐treatment. [file JOCD-25-e70840-s002.jpg]

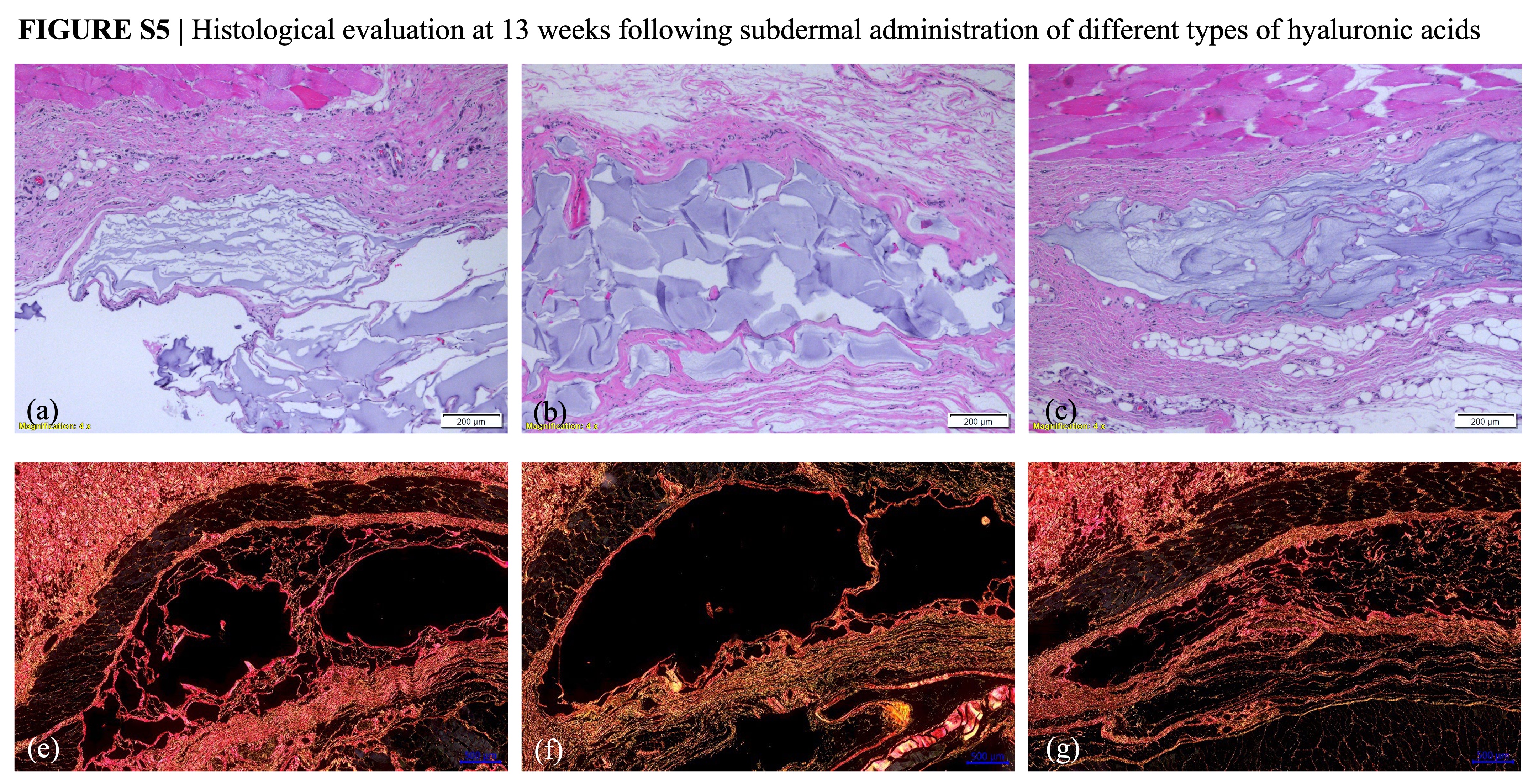

Supplement: Supplementary file 2 — Figure S2: Histological evaluation at 13 weeks following subdermal administration of different types of hyaluronic acids. Representative histological images from a 13‐week subdermal implantation study in Sprague–Dawley rats comparing two commercial hyaluronic acid (HA) fillers (a, b, e, f) and the investigational HLR product (c, g). Top row (a–c): Hematoxylin and eosin (H&E) staining shows tissue architecture and capsule formation around the injected materials. Bottom row (e–g): Picrosirius Red staining under polarized light highlights collagen distribution (types I and III). HLR (c, g) demonstrated superior tissue integration and dispersed collagen deposition within the gel. [file JOCD-25-e70840-s001.jpg]
