# Supplementary material for: Twelve‐Month Outcome of Nasolabial Fold Correction by a Novel Non‐1,4‐Butanediol Diglycidyl Ether, Click‐Crosslinked, Long‐Chain Hyaluronic Acid Product
Source: J Cosmet Dermatol. 2026 Apr 24;25(4):e70840. doi: 10.1111/jocd.70840 (PMC13108564; doi:10.1111/jocd.70840)
Supplement: Supplementary file 3 — Table S1: Detailed Exclusion criteria and wash‐out periods. Table S2: Satisfaction, naturalness questionnaire. Table S3: Satisfaction, GAIS (% of responders as improved or much improved). Table S4: Summary of manufacturer‐provided preclinical biological and tissue‐level evaluation of Hallura products, including rheologic characteristics. [file JOCD-25-e70840-s003.docx]

**Supplementary Data File**

**Table S1. Detailed Exclusion criteria and wash-out periods:**

| - Subject having received (or planning to receive) anti-coagulation, anti-platelet, or thrombolytic medications (e.g., warfarin), anti-inflammatory drugs (oral/injectable corticosteroids or NSAIDs, e.g., aspirin, ibuprofen), or other substances known to increase coagulation time (vitamins or herbal supplements, e.g., vitamin E, garlic, gingko), fish oil or vitamin C from 10 days prior to injection until 3 days post-injection. Study device injections could be delayed as necessary to accommodate this 10-day wash-out period. |
| --- |
| - Subject undergoing one of the following systemic or topical (on the test area) treatments within the specified wash-out periods prior to study start: - antihistamines during the 2 weeks prior to study start - immunosuppressors and/or corticosteroids during the 4 weeks prior to study start - retinoids during the 6 months prior to study start |
| - Subject having received injection with a temporary bioresorbable facial dermal filler (e.g., hyaluronic acid, collagen, autologous fat) within the past 12 months prior to study start. |
| - Subject having received botulinum toxin injections, mesotherapy, or resurfacing procedures (laser, photomodulation, intense pulsed light, radiofrequency, dermabrasion, chemical peel, or other ablative or non-ablative procedures) within 6 months prior to entry into the study. |
| - Subject having received at any time permanent facial implants (e.g., polyacrylamide, PMMA, silicone) anywhere in the face or neck. |

**Table S2. Satisfaction, naturalness questionnaire:**

|  | **Naturalness questionnaire (% of agreement, Response >5 on the likert scale 0 (not at all) – 10 (strongly agree))*** | **Israel Study (HL01IL2022)**  Subject assessment at 12 Months (N=25) |
| --- | --- | --- |
| 1 | I prefer Natural look | 76% |
| 2 | Does the aesthetic result look natural? | 100% |
| 3 | Does the aesthetic result look even? | 96% |
| 4 | Does the aesthetic result look smooth? | 96% |
| 5 | Do you feel that the product is fully integrated in your face? | 100% |
| 6 | How well the treated area feels in your face when you smile? | 100% |
| 7 | How well the treated area feels when your face is relaxed? | 100% |
| 8 | Is the treated area looking more attractive compared to before treatment? | 92% |
| 9 | Is the treated area looking more youthful compared to before treatment? | 92% |
| 10 | Do you feel more confident in yourself compared to before treatment? | 84% |
| 11 | Are you satisfied with the overall treatment experience? | 100% |
| 12 | Are you satisfied with the treatment results? | 96% |
| 13 | Would you likely return for an additional treatment? | 92% |
| 14 | Would you likely be recommending the treatment to a friend? | 100% |

The questionnaire developed in Hallura^®^ and includes 14 statements about different

aspects of perception of the subjects about natural effects of the treatment and satisfaction from the natural results of the treatment. Each statement can be ranked on a 10 Likert scale from 0 to 10 (0-not at all, 10-very satisfied/strongly agree).

**Table S3.** Satisfaction, GAIS (% of responders as improved or much improved)

| **Follow-up post-last treatment** | **Israeli Study**  Physician assessment | **Israeli Study**  Subject assessment | **German and Poland Study**  Physician assessment | **German and Poland Study**  Subject  assessment |
| --- | --- | --- | --- | --- |
| 6 Months | 100% (27/27) | 96% (26/27) | 97% (32/33) | 100% (33/33) |
| 9 Months | 95% (18/19) | 100% (19/19) | 91% (29/32) | 100% (32/32) |
| 12 Months | 100% (25/25) | 100% (25/25) | 94% (31/33) | 97% (32/33) |

Global Aesthetic Improvement Scale (GAIS) ratings at 6, 9, and 12 months post-treatment in the Israeli and German–Poland cohorts. Values indicate the percentage and number of subjects rated as “improved” or “much improved” by physicians and by the subjects themselves. GAIS responses reflect sustained aesthetic satisfaction over time across both cohorts and assessment types.

**Table S4.**

**Summary of manufacturer-provided preclinical biological and tissue-level evaluation of Hallura® products, including rheologic characteristics**

| **1. Ex Vivo Human Skin Explant Study**  **Study Title**: Evaluation of the effects of dermal fillers at three concentrations on human skin explants ex vivo  **Test Facility**: Eurofins BIO-EC, France  **Methods**:   - 12 mm human skin explants from abdominoplasty were injected intradermally with 50 μL of HLR-1 (4 mg/mL), HLR-2 (6 mg/mL), or HLR-3 (8 mg/mL). - Explants were incubated for 7 days at 37°C, then sectioned and processed for histology and immunostaining (collagen I, III, IV, VII, XVII, elastin).   **Results**:   - **Collagen I and Elastin**: Increased expression observed across all HLR products vs. untreated control. - **Collagen III**: Mild increase in HLR-2 and HLR-3. - **Collagen IV, VII, XVII**: Observed upregulation in HLR-1. - **Conclusion**: HLR products stimulated extracellular matrix remodeling, supporting anti-aging and regenerative activity.  \| **% of increase vs non-treated skin** \| **Elastin** \| **Col I** \| **Col III** \| **Col IV** \| **Col VII** \| **Col XVII** \| \| --- \| --- \| --- \| --- \| --- \| --- \| --- \| \| **HLR-1 (4 mg/g)** \| + 104% \| + 14% \| Non specific \| +18% \| +48% \| +39% \| \| **HLR-2 (6 mg/g)** \| + 54% \| + 21% \| + 11% \| not tested \| not tested \| not tested \| \| **HLR-3 (8 mg/g)** \| + 38% \| + 20% \| + 19% \| not tested \| not tested \| not tested \| |
| --- | --- | --- | --- | --- | --- | --- | --- | --- | --- | --- | --- | --- | --- | --- | --- | --- | --- | --- | --- | --- | --- | --- | --- | --- | --- | --- | --- | --- |
| **2. In Vitro Fibroblast Response Study**  **Study Title**: Comparing dermal fibroblast responses to two hyaluronic acid dermal fillers with distinct cross-linking agents: An in vitro study  **Test Facility**: Bioenx srl, Italy  **Methods**:   - Normal human dermal fibroblasts (NHDFs) were exposed to extraction media from HLR-2 or Juvederm Volift for 48 hours. - Supernatants were analyzed by ELISA for collagen I, III, elastin, TGF-β1, IL-1β, and 8-OHdG.   **Results**:   - **Collagen I, III, Elastin**: higher in HLR-2 compared to Juvederm Volift. - **IL-1β**: Comparable across both products, indicating low inflammatory potential. - **8-OHdG**: Lower in HLR-2, suggesting reduced oxidative DNA damage. - **TGF-β1**: Higher in Juvederm Volift. - **Conclusion**: HLR-2 shows superior bio-stimulatory profile and a favorable safety biomarker profile in vitro. |
| **3. Subdermal Implantation in Rats (13-week Study)**  **Study Title**: Histopathological Evaluation Following Single Subdermal Administration of Hyaluronic Acid Products  **Test Facilities**: Pharmaseed, Israel and NAMSA, France  **Methods**:   - Subdermal injection of HLR and commercial fillers (Juvederm Voluma and Restylane with Lidocaine) in Sprague-Dawley rats (100 μL per site, 6 sites per animal). - Histology at Day 95, including H&E, Masson trichrome, Picrosirius Red. - Quantitative image analysis for tissue colonization and capsule thickness.   **Results**:   - **Irritation and Inflammation**: None observed. PII = 0. - **Tissue Integration**: Greater in HLR devices; higher percentage of implant colonized by tissue. - **Capsule Thickness**: Significantly lower in HLR vs. commercial fillers. - **Collagen Distribution**: HLR showed dispersed collagen inside the gel, while commercial fillers had dense fibrotic capsules. - **Conclusion**: HLR devices induced minimal host response and superior integration, suggesting favorable long-term biocompatibility.    C1= Juvederm Voluma, C2= Restylane with Lidocaine, T1= HLR (5 mg/ml), T2= HLR (7.5 mg/ml), T3= HLR (10 mg/ml) |
| **4. Manufacturer-provided rheologic characteristics (G′, G″, and phase angle δ) of HLR-2**  HLR-2 demonstrates unique balance between lifting capacity and tissue softness. Despite a moderate hardness (measured by the elastic modulus alone, G′ ≈ 50 Pa) compared with several established HA-based fillers for the same indication (G′ ranging between 85 – 570 Pa, depending on the crosslinking technology), HLR-2 shows very dominant elastic component (indicated by the ratio between G’ and G’’ and measured by the phase angle δ of 2**°**) compared to the comparators (δ ranging between 4 – 16 **°**). This unique balance between the hardness of a light dermal filler and the elastic response impart HLR-2 with very dynamic lifting capacity, reduced hardness and improved tissue integration in dynamic facial areas.   \| **Sample name** \| **Lot number** \| **Storage Modulus G’ (Pa)** \| **Loss Modulus G’’ (Pa)** \| **Phase angle δ (°)** \| \| --- \| --- \| --- \| --- \| --- \| \| HLR-2 \| H2-N04T04-001 \| 50.10 \| 1.82 \| 2.36 \|   Rheologic data were provided by the manufacturer and derived from internal bench testing. Measurements were performed under standardized oscillatory rheometry conditions. |
